# Single-step genomic evaluation for growth traits in a Mexican Braunvieh cattle population

**DOI:** 10.5713/ab.22.0158

**Published:** 2023-02-28

**Authors:** Jonathan Emanuel Valerio-Hernández, Agustín Ruíz-Flores, Mohammad Ali Nilforooshan, Paulino Pérez-Rodríguez

**Affiliations:** 1 Departamento de Zootecnia, Universidad Autónoma Chapingo, Chapingo, Estado de México, CP 56227, México; 2 Livestock Improvement Corporation, Private Bag 3016, Hamilton 3240, New Zealand; 3 Programa de Estadística, Colegio de Postgraduados, Montecillo, Estado de México. CP 56264, México

**Keywords:** Best Linear Unbiased Prediction (BLUP), Braunvieh, Genomic Selection, genomic BLUP (GBLUP), Single-step Genomic BLUP (ssGBLUP)

## Abstract

**Objective:**

The objective was to compare (pedigree-based) best linear unbiased prediction (BLUP), genomic BLUP (GBLUP), and single-step GBLUP (ssGBLUP) methods for genomic evaluation of growth traits in a Mexican Braunvieh cattle population.

**Methods:**

Birth (BW), weaning (WW), and yearling weight (YW) data of a Mexican Braunvieh cattle population were analyzed with BLUP, GBLUP, and ssGBLUP methods. These methods are differentiated by the additive genetic relationship matrix included in the model and the animals under evaluation. The predictive ability of the model was evaluated using random partitions of the data in training and testing sets, consistently predicting about 20% of genotyped animals on all occasions. For each partition, the Pearson correlation coefficient between adjusted phenotypes for fixed effects and non-genetic random effects and the estimated breeding values (EBV) were computed.

**Results:**

The random contemporary group (CG) effect explained about 50%, 45%, and 35% of the phenotypic variance in BW, WW, and YW, respectively. For the three methods, the CG effect explained the highest proportion of the phenotypic variances (except for YW-GBLUP). The heritability estimate obtained with GBLUP was the lowest for BW, while the highest heritability was obtained with BLUP. For WW, the highest heritability estimate was obtained with BLUP, the estimates obtained with GBLUP and ssGBLUP were similar. For YW, the heritability estimates obtained with GBLUP and BLUP were similar, and the lowest heritability was obtained with ssGBLUP. Pearson correlation coefficients between adjusted phenotypes for non-genetic effects and EBVs were the highest for BLUP, followed by ssBLUP and GBLUP.

**Conclusion:**

The successful implementation of genetic evaluations that include genotyped and non-genotyped animals in our study indicate a promising method for use in genetic improvement programs of Braunvieh cattle. Our findings showed that simultaneous evaluation of genotyped and non-genotyped animals improved prediction accuracy for growth traits even with a limited number of genotyped animals.

## INTRODUCTION

Traditionally, genetic merits of individuals have been predicted estimated breeding value (EBV) using pedigree information [1]. Genomic information can be used to predict EBV. When this occurs, using genomic best linear unbiased prediction (GBLUP) [2], it is called genomic estimated breeding value (GEBV). Genomic selection based on single nucleotide polymorphisms (SNP) accelerates genetic progress by increasing the accuracy of predictions, reducing generation interval, and controlling inbreeding [3].

Nowadays, genomic evaluations are widely used in domestic animal species thanks to the technological advances and a reduction in genotyping costs. Availability of dense SNP panels has made genome-wide or genomic selection evaluation methods effective. Most of the methods for genomic evaluations are usually based on variants of genome-wide association studies for estimating the effects of markers or haplotypes. Differences among the approaches reside in the *a priori* distribution assumed for markers or haplotype effects [4]. However, training these genomic evaluation models generally requires the entire population to be both phenotyped and genotyped, which is not always true or feasible [5]. A more recent method [2] evaluates a population of individuals, not all of which are genotyped. This genomic evaluation method called the single-step genomic BLUP (ssGBLUP) was developed by Christensen and Lund [6] and Aguilar et al [7]. The ssGBLUP method allows the inclusion of both genotyped and non-genotyped animals and uses pedigree and genotype information simultaneously. The two research teams [6,7], using different analytical approaches, arrived at the same formulation for ssGBLUP. The central idea of the method is to create an improved additive genetic relationship matrix (**H**), from the pedigree-based (**A**) and the marker-based (**G**) additive genetic relationship matrices. An advantage of ssGBLUP is that it is extendable to all forms of BLUP by simply replacing the **A**^−1^ matrix in BLUP [1] with the **H**^−1^ matrix, and EBVs are obtained for both genotyped and non-genotyped animals. A statistically equivalent model to ssGBLUP, which we refer to as ssSNPBLUP, was developed by Liu et al [8]. The model directly estimates marker effects, which avoids creating and inverting the genomic relationship matrix. Inverting the genomic relationship matrix can be challenging for a large number of genotyped animals. Liu et al [8] mentioned that the proposed method works well with populations of any size. Other methods equivalent to ssSNPBLUP were developed by Fernando et al [9,10].

The ssGBLUP method has been widely used in both plant and animal breeding [11–13]. Increases in accuracy of predictions by ssGBLUP have been reported, relative to those obtained with BLUP and GBLUP [5,12,14]. The ssGBLUP method has been used in Holstein dairy cattle [15,16], Nordic Red dairy cattle [17], dual-purpose cattle [18], swine [12], turkeys [19], sheep [20,21], and goats [22]. However, to our knowledge the ssGBLUP method has not yet been used in the genetic evaluation of Braunvieh cattle.

In Mexico, BLUP phenotype-based genetic evaluations of Braunvieh cattle are performed on a regular basis [23]. The objective of this study was to compare ssGBLUP, BLUP, and GBLUP, using phenotypic records for growth traits of a Mexican Braunvieh cattle population.

## MATERIALS AND METHODS

### Animals

Phenotypic and pedigree information of the Braunvieh cattle population came from the database of the Mexican Association of Braunvieh Purebreeders (Asociación Mexicana de Criadores de Ganado Suizo de Registro, Mexico City, Mexico). Birth weight (BW), weaning (at 218 days of age) weight (WW), and yearling weight (YW) phenotypes were used. Data on 10 generations back were extracted. Animals were born from 1998 to 2016.

For the genomic information, hair samples were col lected from 300 animals in eight herds located in Eastern, Central, and Western Mexico. The samples were genotyped at GeneSeek (Lincoln, NE, USA; https://www.neogen.com/), using the GeneSeek Genomic Profiler Bovine LDv.4 panel, with 30,000 and 50,000 SNP markers, 150 animals with each Chip.

### Phenotypes

Data editing for BW started with 32,159 records. Outliers were removed (animals with phenotypes higher or lower than the average phenotype in the database ±3 standard deviations). Records from animals without information of herd or dam age were deleted as well. Animals not genetically related to the 300 genotyped animals were discarded because the solutions for non-genotyped animals not related to genotyped animals would remain the same between BLUP and ssGBLUP. Contemporary groups (CG) were defined by combining the effects of herd (8 herds), year (from 1998 to 2016), and season of birth. Birth seasons were defined considering the Julian calendar, from 80 to 171 d, spring; from 172 to 264 d, summer; from 265 to 354 d, autumn; from 355 to 366 d, and from 1 to 79 d, winter. The days were counted continuously from the beginning of the Julian period, starting from the first of January each year. Records of animals in CG with fewer than two animals or in CG with variance equal to zero were also discarded. Data edition resulted in 330 BW records for further analysis. Of the phenotyped animals, 232 had been genotyped.

For WW data, editing started with 29,142 records. Those records with erroneous age at weaning, were outside the range average ±3 standard deviations, or had no information on management, herd, or age of dam were discarded. Animals not genetically related to the 300 genotyped animals were deleted as well. Contemporary groups for WW were defined in the same way as for BW with 6 herds and birth years from 1998 to 2016. Three management groups were defined: calves fed milk from their dam, calves fed milk from their dam plus balanced feed, and calves fed milk from their dam and a wet nurse plus balanced feed. Records of animals in CG with fewer than two animals or in CG with variance equal to zero were also discarded. At the end of data editing, 267 WW records were left for further analysis. Of the phenotyped animals, 218 had been genotyped.

Data editing for YW started with 19,971 records. Those with discordant age, or outside the average ±3 standard deviations, with no herd or management information were deleted. Animals not genetically related to the 300 genotyped animals were discarded as well. Contemporary groups were defined in the same way as for WW. Three management groups for YW were defined: animals kept under grazing, animals that grazed and received balanced feed, and confined animals that received balanced feed. Records of animals in CG with fewer than two animals or in CG with variance equal to zero were also discarded. At the end of data editing, 232 records for YW were available for subsequent analyses. Of the phenotyped animals, 191 had been genotyped.

### Genotypes

Data from 300 genotyped animals (236 females and 64 males) born between 2001 and 2016 were used. The SNPs in common between the 30K and 50K Chips were used (12,835 SNPs). Proportions of missing values for each marker and for each individual were calculated. The average of missing values by individual was 2.09% with a standard deviation of 7.50%. The average call rate (non-missing proportion for each marker) was 97.90% with a standard deviation of 4.66%. Markers with a call rate below 95% were removed. Marker genotypes were recoded for additive effects as 0, 1, 2 (aa = 0, Aa or aA = 1, and AA = 2). Missing genotypes were imputed using 2*θ̂_j_*, where *θ̂_j_* is the estimated frequency of the allele coded as one at the j-th marker [24,25]. Monomorphic markers or those with minor allele frequency less than 0.04 were removed. After all the cleaning and quality control process, 11,646 of the 12,835 SNPs in common between the two Chips were available for further analysis.

### Calculation of the genetic relationship matrices

The inverse of the additive genetic relationship matrix (**A**^−1^) was obtained from the pedigree information, using the function ‘getAInv’ from R package ‘pedigreemm’ [26]. The genomic relationship matrix was calculated as described by López-Cruz et al [27] and Pérez-Rodríguez et al [13]. Briefly, **G** = **WW′**/q, where **W** is the matrix of recoded marker genotypes centered and standardized by column, and *q* is the number of markers. Because all markers were standardized to a variance of one, the average of the diagonal elements of **G** was about one, so that both matrices **A** and **G** have similar scaling.

The inverse of the combined pedigree and genomic addi tive relationship matrix, including genotyped and non-genotyped animals (**H**^−1^), is obtained directly without the need to form **H**. This matrix is defined as [7,12]:


$$H^{-1}=A^{-1}+\left[\begin{array}{cc} 0 & 0\\ 0 & G_{a}^{-1}-A_{gg}^{-1} \end{array}\right],$$

where, **A***_gg_* is the submatrix of **A** for genotyped animals, **G***_a_* = *β***G**+*α*; *β* and *α* were obtained by solving the equations:


$$\{\begin{array}{c} Avg(diag(G))\beta +\alpha =Avg\left(diag(A_{gg})\right)\\ Avg(G)\beta +\alpha =Avg(A_{gg}) \end{array}.$$

The obtained *α* (0.0296) and *β* (0.9810) values set the mean of **G***_a_* to the mean of **A***_gg_*, and the mean of diag(**G***_a_*) to the mean of diag(**A***_gg_*). The scaling for the matrices **G** and **A** is similar, and as mentioned before the mean is around one, this is important since those matrixes are used as variance covariance matrixes that are used to fit models and compute variance components that can be difficult to interpret if matrixes are not scaled correctly. The diagonal entries of **G** matrix ranges from 0.50 to 1.27, whereas for matrix **A** diagonal entries ranges from 1.00 to 1.26, for matrix **H**, the diagonal entries ranges from 0.51 to 1.34 and the average of these elements is also around 1.

### Statistical analysis

Growth traits BW, WW, and YW were analyzed with different univariate models and three alternative methods, BLUP, GBLUP, and ssGBLUP. The most important difference among these methods is the inverse relationship matrices used (**A**^−1^, **G**^−1^, **H**^−1^) and the animals under evaluation (only genotyped animals for GBLUP).

Analyses of the three traits were performed with the linear model:


(1) $$y=X\beta +Z_{1}c+Z_{2}a+e$$

where ***y*** is the vector of phenotypes, **X** is the incidence matrix relating phenotypes to fixed effects (3, 4, and 4 fixed effects for BW, WW, and YW), **Z**_1_ is the incidence matrix relating phenotypes to random CGs (54, 43, and 37 CG for BW, WW, and YW in GBLUP, and 96, 66, and 59 CG for BW, WW, and YW in BLUP and ssGBLUP), **Z**_2_ is the incidence matrix relating phenotypes to random additive genetic effects, and e is the vector of random residual effects. Additive genetic, CG, and residual variances were $Var(a)=\Delta \sigma_{a}^{2},Var(c)=I\sigma_{cg}^{2}$, and $Var(e)=I\sigma_{e}^{2}$, respectively. Matrix **Δ** is **H**, **A**, or **G**, depending on the method of analysis, and $\sigma_{a}^{2},\;\sigma_{cg}^{2}$, and $\sigma_{e}^{2}$ are the corresponding variances. Variance components for each method were estimated via the R package ‘BGLR’ [28] using the Bayesian framework. Hyper-parameters were set automatically according to internal rules [28]. Inferences were based on 25,000 MCMC iterations obtained after discarding 25,000 samples taken as burn-in and with a thin set to 10. The covariate age of the dam in days and sex were considered as fixed effects for BW; for WW age of dam, sex, management group and the covariate of weaning age in days; similarly, for YW age of dam, sex, yearling age, and management group were considered as fixed effects. Weaning age and yearling age were treated as continuous covariates.

### Validation analysis

#### GBLUP model

To study the predictive ability of the models, we partitioned the data at random to 80% of the observations as the training set and the remaining 20% as the testing set. We generated 100 partitions of the data at random and fitted model (1) using the BGLR package. The same parameters and hyperparameters described in the previous section were used for the variance components estimation. For each partition, we computed the Pearson correlation coefficient between the phenotypes adjusted for fixed effects and non-genetic random effects and EBV. The average of the Pearson correlation coefficients and their standard deviations were calculated. For each of the 100 random validation partitions, we kept a record of which individuals were assigned to the testing set to validate the same set of individuals with BLUP and ssGBLUP methods.

#### BLUP and ssGBLUP methods

To study the predictive ability of these methods, the data was partitioned into training and testing subsets. We assigned the same individuals predicted with the GBLUP model, as well as the rest of the individuals, to the testing set to have a common set of individuals whose breeding values needed to be predicted. We fitted the models and calculated Pearson correlation coefficients between the adjusted phenotypes and the EBVs using the same strategy as that used in the GBLUP model.

## RESULTS

Table 1 shows the estimates of additive genetic ( $\sigma_{a}^{2}$), CG ( $\sigma_{cg}^{2}$) and residual variances for BW, WW, and YW. The random CG effect explained about 50%, 45%, and 35% of the phenotypic variance for BW, WW, and YW, respectively. For the three traits and the three methods, $\sigma_{cg}^{2}$ explained the highest proportion of the phenotypic variance, except for YW-GBLUP. Heritability estimates (h^2^) are also presented in Table 1. For BW, the heritability estimate obtained with GBLUP was the lowest, while the highest was obtained with BLUP. For WW, the highest heritability estimate was obtained with BLUP; the estimates obtained with GBLUP and ssGBLUP were similar. For YW, the heritability estimates obtained with GBLUP and BLUP were similar; the lowest was obtained with ssGBLUP. Within traits, the heritability estimates obtained with the three alternative methods were within a narrow range, except for BW-GBLUP (h^2^ = 0.128). The high standard errors associated with the variance components (Table 1) might be due to the small sample size.

**Figure 1:**
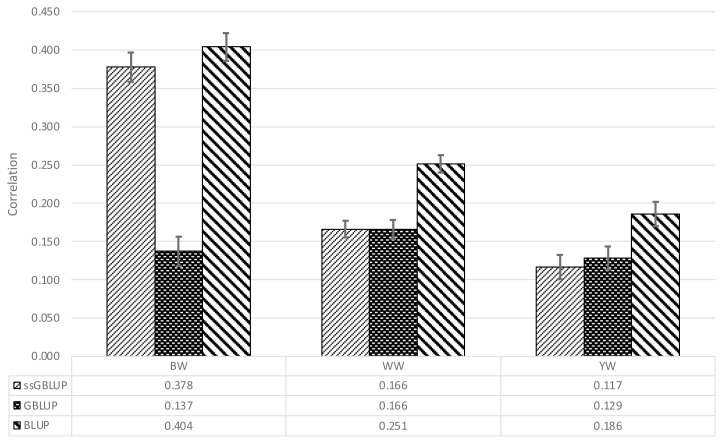
Pearson correlation coefficients between corrected phenotypes and the estimated breeding value obtained with three different models for birth (BW), weaning (WW), and yearling weight (YW) of a Mexican Braunvieh cattle population.

Table 2 shows the Pearson correlation coefficients between the adjusted phenotypes and the EBVs. For BW, the lowest and the highest correlations were obtained with GBLUP and BLUP, respectively. BLUP yielded consistently higher estimates than either GBLUP or ssGBLUP. Figure 1 shows graphically the results of the validation; each bar represents a correlation estimate and the error bars represent the associated standard errors computed by dividing the standard deviation in parenthesis in Table 2 by 10, the square root of the number of random partitions evaluated.

## DISCUSSION

A potential benefit of ssGBLUP over BLUP and GBLUP is that it utilizes data used in both BLUP and GBLUP, simultaneously. Thus, ssGBLUP and BLUP use phenotypes and pedigrees from genotyped and non-genotyped animals. As GBLUP is limited to genotyped animals, only phenotypes of genotyped animals were used.

The statistical models for BW and WW typically include a maternal genetic effect. However, we did not have enough information to estimate this effect to include it in the model due to the small sample size. Consequently, this will lead to an increase in the uncertainty of predictions.

For the three traits and the three methods tested, $\sigma_{cg}^{2}$ had the greatest magnitude among the estimated variance components. Among all the trait-method combinations, $\sigma_{a}^{2}$ was always the greatest with BLUP (Table 1). In contrast, $\sigma_{a}^{2}$ was consistently the lowest for GBLUP. Estimation of the variance components and genetic parameters is useful for planning selection in breeding programs; additive genetic variance has long been regarded as the most important.

The heritability estimates were within the range of what has been reported in other studies. For example, heritability estimates for several cattle breeds using BLUP (**A**^−1^) were in ranges from 0.15 to 0.36, from 0.10 to 0.27, and from 0.18 to 0.30 for BW, WW and YW, respectively [29,30].

Pearson correlation estimates between the adjusted phe notypes and the EBVs were highest for BLUP, followed by ssGBLUP. The estimates obtained with GBLUP and ssGBUP were quite similar for YW. The reported Pearson correlation coefficients between corrected phenotypes and EBVs allow us to determine how well a model predicts the additive genetic merits. In this study, BLUP gave the best predictions. We also calculated Pearson correlation coefficients between observed and predicted phenotypes. This correlation determines the ability of the model to predict the phenotype of interest, given the information provided in the model. Other studies [12,31,32] reported better model fits by including genomic information; however, genomic information in this study was limited.

Table 2 presents the Pearson correlation coefficients be tween corrected phenotypes and EBVs together with the standard errors around the correlation coefficients. The number of individuals in the training set for methods BLUP and ssGBLUP was larger than in the training set for GBLUP, and the number of individuals predicted in the three cases were the same. The sample size in the training set could result in better predictions in the testing set when datasets are small.

Other studies obtained higher prediction accuracies using ssGBLUP [11,12,14,19]. However, these authors used larger populations than that of our study. We used 11,648 SNPs to compute **G** and **H** matrices, while in the mentioned studies 53,455, 45,818, 25,720, and 57,000 SNP markers, respectively, were used. Even though, the number of SNPs was not as large as in those studies, the correlation estimates results (BLUP vs ssGBLUP) suggest that the information provided by 11,648 markers is valuable.

Christensen et al [12] studying average daily gain and feed conversion in Danish Duroc swine, reported that ssGBLUP and GBLUP yielded more accurate predictions than BLUP. These advantages held true in both uni- and bivariate analyses. Additionally, they found that the ssGBLUP method resulted in more accurate predictions for non-genotyped animals [12]. These authors concluded that ssGBLUP yields more accurate predictions for genotyped animals than BLUP, and similar accuracy compared with GBLUP for genotyped animals. Montes et al [29] and Park et al [32] reported slightly higher accuracies for ssGBLUP than for BLUP for five carcass traits of Hanwoo cattle.

## CONCLUSION

The literature reports that ssGBLUP outperforms pedigree and marker-based methods. The gains in some cases are slightly higher and depend on several factors, for example, number of individuals genotyped and non-genotyped, sample size, number of markers, and heritability of the traits. In our case, the number of genotyped animals was small compared with other studies, and this is perhaps the main reason that we did not observe a clear advantage of ssGBLUP over BLUP. However, the results are encouraging, and we will continue genotyping and phenotyping more animals (especially those with more relatives in the population) in the years to come to increase our sample sizes. Another possibility for improving the benefits of ssGBLUP is making use of genotypes on a larger set of markers by genotyping more animals for both 30K and 50K panels to make imputation to the 50K panel possible.

## Figures and Tables

**Table 1:** Estimates of variances and heritability for birth weight, weaning weight, and yearling weight of a Mexican Braunvieh cattle population obtained after fitting three different models

Items	Component	GBLUP			BLUP			ssGBLUP		
		
		Value	SE	%	Value	SE	%	Value	SE	%
BW	$\sigma_{a}^{2}$	1.813	0.554	12.88	3.323	0.859	26.02	3.213	0.835	24.49
	$\sigma_{cg}^{2}$	9.21	2.312	65.42	6.348	1.531	49.71	6.791	1.552	51.77
	$\sigma_{e}^{2}$	3.056	0.517	21.70	3.099	0.585	24.27	3.115	0.594	23.74
	h^2^	0.128			0.260			0.244		
WW	$\sigma_{a}^{2}$	137.780	51.824	19.25	231.475	84.339	22.35	206.365	75.399	19.57
	$\sigma_{cg}^{2}$	297.556	94.466	41.58	465.301	139.124	44.94	490.046	140.217	46.47
	$\sigma_{e}^{2}$	280.373	50.549	39.17	338.71	68.278	32.71	358.199	66.169	33.97
	h^2^	0.192			0.223			0.195		
YW	$\sigma_{a}^{2}$	428.235	175.700	22.16	612.485	220.039	23.16	524.815	200.708	19.37
	$\sigma_{cg}^{2}$	631.958	212.973	32.70	1,097.47	365.987	41.50	1,187.73	379.402	43.82
	$\sigma_{e}^{2}$	872.358	168.841	45.14	934.302	194.968	35.33	997.386	187.932	36.81
	h^2^	0.221			0.231			0.193		

GBLUP, genomic best linear unbiased prediction; BLUP, best linear unbiased prediction; ssGBLUP, single-step GBLUP; SE, standard errors for the variance parameters; BW, birth weight; WW, weaning weight; YW, yearling weight; σ_a^2, additive genetic variance; σ_cg^2, contemporary group variance; σ_e^2, residual variance; h^2^, heritability.

**Table 2:** Pearson correlation (Cor) and standard deviation (SD) estimates between corrected phenotypes and estimated breeding values obtained from cross validation with BLUP, GBLUP and ssGBLUP models, for BW, WW, YW

Item	BW		WW		YW	
		
	Cor	SD	Cor	SD	Cor	SD
GBLUP	0.137	0.190	0.166	0.126	0.129	0.150
BLUP	0.404	0.180	0.251	0.114	0.186	0.153
ssGBLUP	0.378	0.194	0.166	0.112	0.117	0.160

BW, birth weight; WW, weaning weight; YW, yearling weight; Cor, correlation; SD, standard deviation; GBLUP, genomic best linear unbiased prediction; BLUP, best linear unbiased prediction; ssGBLUP, single-step GBLUP.
